# Comprehensive genomic analysis of microenvironment phenotypes in ovarian cancer

**DOI:** 10.7717/peerj.10255

**Published:** 2020-11-23

**Authors:** Jingshu Wang, Tingting Zhang, Lina Yang, Gong Yang

**Affiliations:** 1 Central Laboratory, The Fifth People’s Hospital of Shanghai, Fudan University, Shanghai, China; 2 Acupuncture and Tuina College, Shanghai University of Chinese Traditional Medicine, Shanghai, China; 3 Department of Gynecological Oncology, The Fifth People’s Hospital of Shanghai, Fudan University, Shanghai, China; 4 Cancer Institute, Fudan University Shanghai Cancer Center, Fudan University, Shanghai, China; 5 Department of Oncology, Shanghai Medical School, Fudan University, Shanghai, China

**Keywords:** Ovarian cancer, Immune infiltration, Tumor microenvironment, Random forest, Immunotherapy

## Abstract

**Background:**

Ovarian cancer is one of the leading causes of cancer-related death in women. The incidence of ovarian cancer is insidious, and the recurrence rate is high. The survival rate of ovarian cancer has not significantly improved over the past decade. Recently, immune checkpoint inhibitors such as those targeting CTLA-4, PD-1, or PD-L1 have been used to treat ovarian cancer. Therefore, a full analysis of the immune biomarkers associated with this malignancy is necessary.

**Methods:**

In this study, we used data from The Cancer Genome Atlas (TCGA) database to analyze the infiltration patterns of specific immune cell types in tumor samples. Data from the Gene Expression Omnibus (GEO) database was used for external validation. According to the invasion patterns of immune cells, we divided the ovarian cancer microenvironment into two clusters: A and B. These tumor microenvironment (TME) subtypes were associated with genomic and clinicopathological characteristics. Subsequently, a random forest classification model was established. Differential genomic features, functional enrichment, and DNA methylation were analyzed between the two clusters. The characteristics of immune cell infiltration and the expression of immune-related cytokines or markers were analyzed. Somatic mutation analysis was also performed between clusters A and B. Finally, multivariate Cox analysis was used to analyze independent prognostic factors.

**Results:**

The ovarian cancer TME cluster A was characterized by less infiltration of immune cells and sparse distribution and low expression of immunomodulators. In contrast, cytotoxic T cells and immunosuppressive cells were significantly increased in the ovarian cancer TME cluster B. Additionally, immune-related cytokines or markers, including IFN-*γ* and TNF-*β*, were also expressed in large quantities. In total, 35 differentially methylated and expressed genes (DMEGs) were identified. Functional enrichment analyses revealed that the DMEGs in cluster B participated in important biological processes and immune-related pathways. The mutation load in cluster B was insignificantly higher than that of cluster A (*p* = 0.076). Multivariate Cox analysis showed that TME was an independent prognostic factor for ovarian cancer (hazard ratio: 1.33, 95% confidence interval: 1.01–1.75, *p* = 0.041).

**Conclusion:**

This study described and classified basic information about the immune invasion pattern of ovarian cancer and integrated biomarkers related to different immunophenotypes to reveal interactions between ovarian cancer and the immune system.

## Introduction

Epithelial ovarian cancer is the main pathological type of ovarian cancer, and it has some histological subtypes, such as such as ovarian clear cell carcinoma, mucinous ovarian cancer and serous ovarian cancer (Lowe et al., 2013). Global results showed a wide range in ovarian cancer incidence rates, and ovarian cancer survival has shown modest improvement from a statistical perspective in the U.S (Barnholtz-Sloan et al., 2003; Lowe et al., 2013; Park, Ruterbusch & Cote, 2017). The efficacy of immunotherapy can be further improved in ovarian cancer by analyzing the immune indexes of these patients to predict their responses to immune checkpoint blockade. The tumor microenvironment (TME) consists of a variety of innate and adaptive immune cells and a series of cytokines that regulate their responses to tumor cells. Solid tumors interact with infiltrating immune cells and inflammatory molecules in a dynamic equilibrium that affects disease development and outcome. Therefore, recently, several researches have investigated the functions of immune cells and cytokines in the TME in tumor development and/or tumor immune escape. These studies have also reported new methods of evaluating the infiltration of immune cells or factors in the TME (Lorenzo-Sanz & Munoz, 2019). These studies have proposed several immune signals that control the immune infiltration pattern of the TME, which can be used to accurately screen the immune responses to gastric cancer, non-small cell lung carcinoma (NSCLC) and other malignant tumors, and are potential therapeutic candidates (Bi et al., 2020).

In this study, we used the gene expression profile data of 782 ovarian cancer patients, including all histological subtypes, from The Cancer Genome Atlas (TCGA) and Gene Expression Omnibus (GEO) databases. According to the immune cell invasion and cytokine expression profiles of the TME, patients were divided into two different TME clusters. We found that the survival rate of patients in the low infiltration group (TME cluster A) was higher than that of the high infiltration group (TME cluster B). We also integrated biomarkers related to different immunophenotypes and analyzed genes with differential methylation levels, which provided evidence to better understand the interaction between ovarian cancer and the immune system. Our study aimed to develop an immune pattern and identify the immune signature of ovarian cancer.

## Methods and Materials

### Ovarian cancer expression data sets

We downloaded the level-4 mRNA sequencing data of ovarian cancer patients (presented as FPKM) and the related clinical data from TCGA database; these data were used as the discovery cohort. Patients were removed if their clinical and/or survival information were vague or unavailable. Additionally, we collected and enrolled the ovarian cancer patients’ microarray data from several independent datasets in the GEO database, including GSE3149, GSE26712, and GSE63885. These gene expression data were all generated and annotated by platform Affymetrix Human Genome U133 Plus 2.0 (GPL570) or Affymetrix Human Genome U133a (GPL96). The COMBAT experience empirical Bayes approach in the *sva* package was used to eliminate the heterogeneous batch effect among different studies (Johnson, Li & Rabinovic, 2007). The background was adjusted and the data were normalized using the *limma* package (Ritchie et al., 2015).

### Estimation and classification of TME cell abundances

We comprehensively analyzed and identified the published literature and ultimately utilized the gene signatures to build a compendium of microenvironment-associated genes (Bindea et al., 2013). The gene signature included 585 genes and represented 24 microenvironment cell subtypes from both innate and adaptive immunity, namely T cells, cytotoxic cells, CD8 T cells, as well as T*γδ*, T helper, Tcm, Tem, Th1, Th2, Th17, Tfh, Tgd, and Treg cells, B cells, eosinophils, macrophages, mast cells, neutrophils, dendritic cells (DCs), immature DCs, activated DCs (aDCs), natural killer (NK) cells, NK CD56-dim cells, and NK CD56-bright cells. The relative enrichment level of each immune cell subset was estimated based on the RNA expression data of each ovarian cancer patients using the Gene Set Variation Analysis (GSVA) algorithm in the *GSVA* package (Hänzelmann, Castelo & Guinney, 2013), which is a non-parametric and unsupervised analysis method that is primarily used to evaluate the gene set enrichment of nuclear transcriptome microarray data. It has been reported that GSVA outperforms single cell Gene Set Enrichment Analysis (ssGSEA) when calculating the signal-to-noise ratio in differential gene expression and differential signaling pathways because GSVA included normalization of gene expression to reduce the noise of the data (Tamborero et al., 2018). Finally, we obtained enrichment scores ranging from −1 to 1 for those 24 immune cell types, which indicated the relative enrichment of the immune cell infiltration.

An unsupervised clustering approach on the basis of Euclidean distance and Ward’s linkage was used to cluster the ovarian cancers with distinct immune cell infiltration patterns. We use the *ConsensusClusterPlus* package containing 1,000 repeats to determine the optimal number of clusters for the “Infiltrated group” based on the percentage of data variance.

We determined the optimal clustering number of the “Infiltration group” on the basis of the percentage of variance of the data using the *ConsensusClusterPlus* package with 1,000 repeats (Monti et al., 2003). The distribution patterns of the 24 immune cell subtypes in each patient were produced by utilizing the *pheatmap* package.

### Differential genomic features and functional enrichment analysis

The *limma* package was adopted to screen differentially expressed genes (DEGs) and miRNAs in different infiltration groups. The *limma* software package used Benjamin and Hochberg (BH) methods to estimate the gene expression profile through the moderation *t*-test. The *p*-value was adjusted to the false discovery rate (Ritchie et al., 2015). —Log (fold change) (logFC) >0.5 with adjusted *p* < 0.05 were considered the cut-off criteria for differential expression. The cluster analysis software package was adopted to analyze the functional enrichment of DEGs (Yu et al., 2012). Both Gene Ontology (GO) and Kyoto Encyclopedia of Genes and Genomes (KEGG) terms were identified strictly at *p* < 0.05. Additionally, the *maftools* software package based on the Kruskal–Wallis test was used to measure the mutation distribution of somatic cells and copy number variation detected by gistic. An adjusted *p*-value <0.01 was regarded to be significant.

### Analysis of DNA methylation data

We employed the TCGA DNA methylation obtained by the Illumina Human Methylation450 BeadChip array, which contains 485577 probes (approximately 396066 after filtering invalid probes) and covers 99% of RefSeq genes. The methylation level of the probe was quantified as *β*-values, which were defined as the ratio of the strength of the methylated and unmethylated alleles. The Champ pipeline was used to normalize 5′-c-phosphate-g-3′ (CPG) methylation data of different infiltration groups, and the results were compared with those of DEG analysis. The threshold value for CpG sites was set as a corrected *p*-value of <0.05 and an absolute Δ*β*-value of >0.2. In this study, when the number of differentially methylated CPGs in the promoter region of a gene was >1, it was considered a differentially methylated gene. We focused on the methylation of gene promoter regions, including TSS200, TSS1500, T′-UTR, and 1st-Exon, since differentially methylated genes (DMGs) are commonly defined according to their promoter methylation status (Lee et al., 2004; Licchesi et al., 2008; Liu et al., 2008). According to both DMGs and DEGs, genes were classified into four categories: hypermethylated-upregulated (hyper-up), hypermethylated-downregulated (hyper-down), hypomethylated-upregulated (hypo-up), and hypomethylated-downregulated (hypo-down).

### Establishing of the random forest classification model

Establishing the random forest classification model depended on the random forest algorithm and the random forest packets of thousands of classification trees or regression trees. First, we randomly divided the ovarian cancer patients in TCGA into training and experimental cohort at a 7:3 ratio. The number of variables (mtry) and the number of trees to grow (ntrees) were randomly allocated. The optimal value for the training group to achieve the lowest error rate was determined after 10,000 iterations. According to the above parameters, a random forest classification model was constructed. Additionally, the variable importance (VIMP) value was set to measure the prediction error rate of the model.

### Statistical analysis

All statistical data and images were analyzed and plotted by R version 3.5.3 (R Foundation for Statistical Computing, Vienna, Austria) (Colaprico et al., 2016). Continuous variables between the high- and low-invasiveness groups were compared by Student’s *t*-test and the Wilcoxon test. Categorical variables were analyzed by the chi-square test. Kaplan–Meier analysis and log-rank tests were performed using the *ggplot2* package to analyze overall survival (OS). To estimate the prognostic value of invasion by different immune cell subtypes, the *survminer* software package was used to identify the cutoff value based on the correlation between OS and GSVA scores. Finally, the *forestplot* package was used to present the results. Two-sided *p*-value of <0.05 were regarded as statistically significant in the chi-square test, correlation test, log-rank test, and Cox analysis.

## Results

### Phenotypic landscape of the ovarian cancer microenvironment

**Figure 1:**
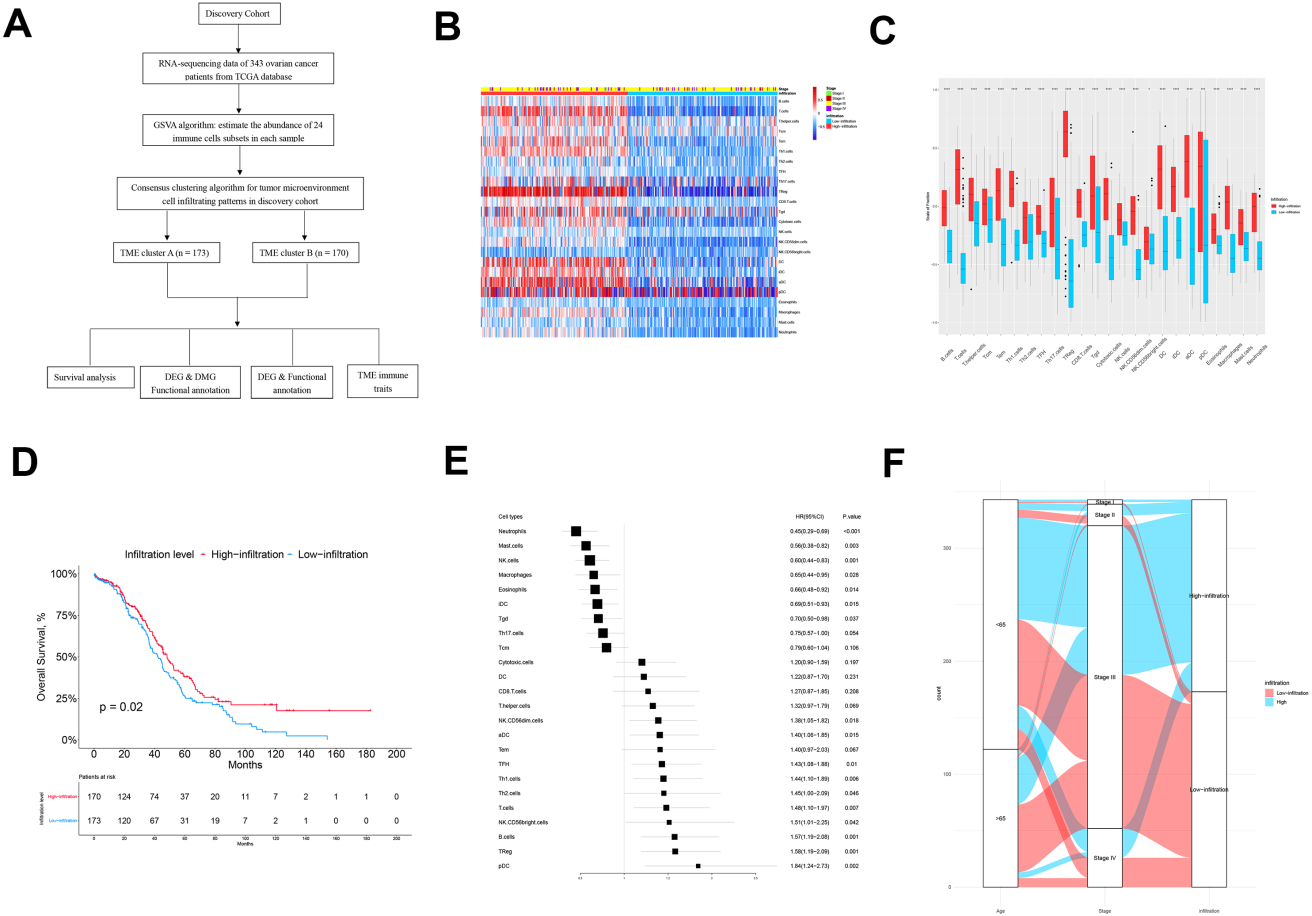
The TME clusters of ovarian cancer from TCGA cohort. (A) Flowchart of the selection process. (B) Unsupervised clustering of tumor microenvironment immune cells for 343 patients from TCGA. The statistical difference of two TME clusters was compared using the Wilcoxon test. (C) The fraction of immune cells in TME cluster A and B in patients from TCGA. (D) Kaplan–Meier curves revealed that the patients with high infiltration had better OS than those with low infiltration (*p* = 0.02). (E) Forest plot showing the prognostic value of each type of immune cell. (F) The distribution of baseline information in TME cluster A and B.

The flow chart of this study is shown in Fig. 1A. There were 343 patients from TCGA enrolled in the discovery cohort and 439 patients from the GEO database enrolled in the validation cohort. We used a GSVA enrichment fraction containing multiple immune cell types to describe the relative abundance and comprehensively describe the immune invasion phenotype of each ovarian cancer patient. K-means clustering divided the 343 patients into TME cluster A (*n* = 170) and TME cluster B (*n* = 173) according to the dichotomy of the discovery cohort (TCGA data). The different groups reviewed the different immune infiltration patterns of each ovarian cancer patient’s adaptive and innate immune system (Fig. 1B). TME cluster A was featured with less abundance of immune cells and a sparse distribution and low expression of immunomodulators. In contrast, the activation levels of innate and/or adaptive immune responses in TME cluster B were relatively high, indicating high infiltration of T cells, B cells, Tregs, TGD cells, cytotoxic cells, DCs, IDCs, ADCs, and macrophages (Figs. 1B and 1C).

We estimated the OS of two the TME clusters, and the survival rate of patients in the low infiltration group (TME cluster A) was higher than of those in the infiltration group (TME cluster B) (*p* = 0.02; Fig. 1D). Due to the prognostic impact of TME cluster, we next investigated the relationship between each immune cell type and prognosis. According to the forest map, most tumor infiltrating immune cells were associated with improved OS, including NK Cd56-dim cells (*p* = 0.018), ADCs (*p* = 0.015), TFHs (*p* = 0.01), Th1 cells (*p* = 0.006), Th2 cells (*p* = 0.046), T cells (*p* = 0.007), NK CD56-bright cells (*p* = 0.042), B cells (*p* = 0.001), Tregs (*p* = 0.001), and PDCs (*p* = 0.002) (Fig. 1E); the exceptions were neutrophils (*p* < 0.001), mast cells (*p* = 0.003), NK cells (*p* = 0.001), macrophages (*p* = 0.028), eosinophils (*p* = 0.014), IDCs (*p* = 0.015), and TGDs (*p* = 0.037). These results showed that the TME group B had more immune cell infiltration than TME group A patients, which was consistent with improved OS.

Next, we explored the distribution of age and tumor stage between TME clusters A and B (Fig. 1F and Table 1). The results showed that the level of immune infiltration in elderly patients was lower than that of young patients (*p* = 0.046). No significant difference was observed regarding the distribution different tumor stages among the two TME clusters (*p* = 0.994).

**Table 1:** Baseline characteristics of the OV patients in the two infiltration-groups from TCGA database.

Characteristics	High-Infiltration (*n* = 170)	Low-Infiltration (*n* = 173)	*P*-value
Age			
≤65	125 (%)	109 (27.2%)	0.048
>65	45 (%)	64 (34.9%)	
Stage			
Stage I	2 (53.7%)	2 (55.9%)	0.994
Stage II	10 (24.0%)	9 (25.0%)	
Stage III	132 (15.6%)	136 (15.8%)	
Stage VI	26 (6.7%)	26 (3.3%)	

### Establishing and verification of the random forest classification model

To establish a practical clinical method, we divided ovarian cancer patients into TME cluster A and TME cluster B, and then established a forest classification model according to the Breiman random forest algorithm. As described below, 343 patients from the TCGA database were randomly allocated into a training group (*n* = 240) and an experimental group (*n* = 103). The relative abundance (GSVA score) of the 24 immune cell types was used as the input variable, and TME cluster determined by unsupervised clustering was used as response factor. In the training queue, after 10,000 iterations, the optimal mtry and ntrees were 4 and 4000, respectively. To test the discriminatory ability of the model, we applied it to the training queue and internal test queue to verify the effectiveness of the model. The prediction accuracy was 100% and 99%, respectively, which proved the reliability and stability of the model.

**Figure 2:**
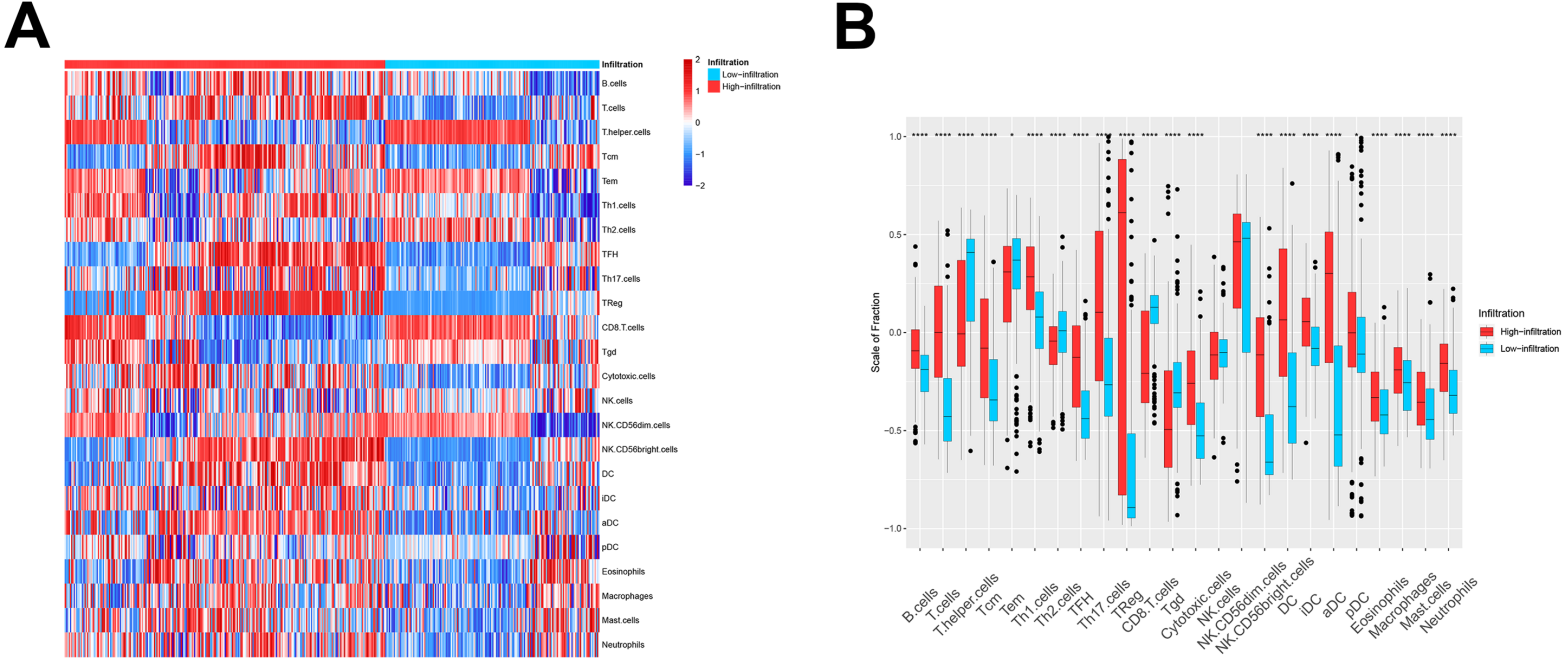
The TME cluster of ovarian cancer in GEO cohort. (A) The relative abundance of 24 types of immune cells in 493 ovarian cancer patients. (B) The fraction of immune cells in TME cluster A and B patients from GEO.

We then used these models in the external validation cohort from the GEO database, which included 493 ovarian cancer patients (GSE3149, GSE26712, and GSE63885). After GSVA scores of 2, the four immune cell types were calculated and fitted with the model, the patients were divided into TME cluster A and B for external validation. The heatmap (Fig. 2A) and box plot (Fig. 2B) revealed that TME cluster B from the GEO database was also enriched for cells of the innate and adaptive immune systems, which was consistent with analysis of TCGA data. These results further confirmed the clinical and immunological significance of the random typing model.

### Identification and functional annotation of DEGs

**Figure 3:**
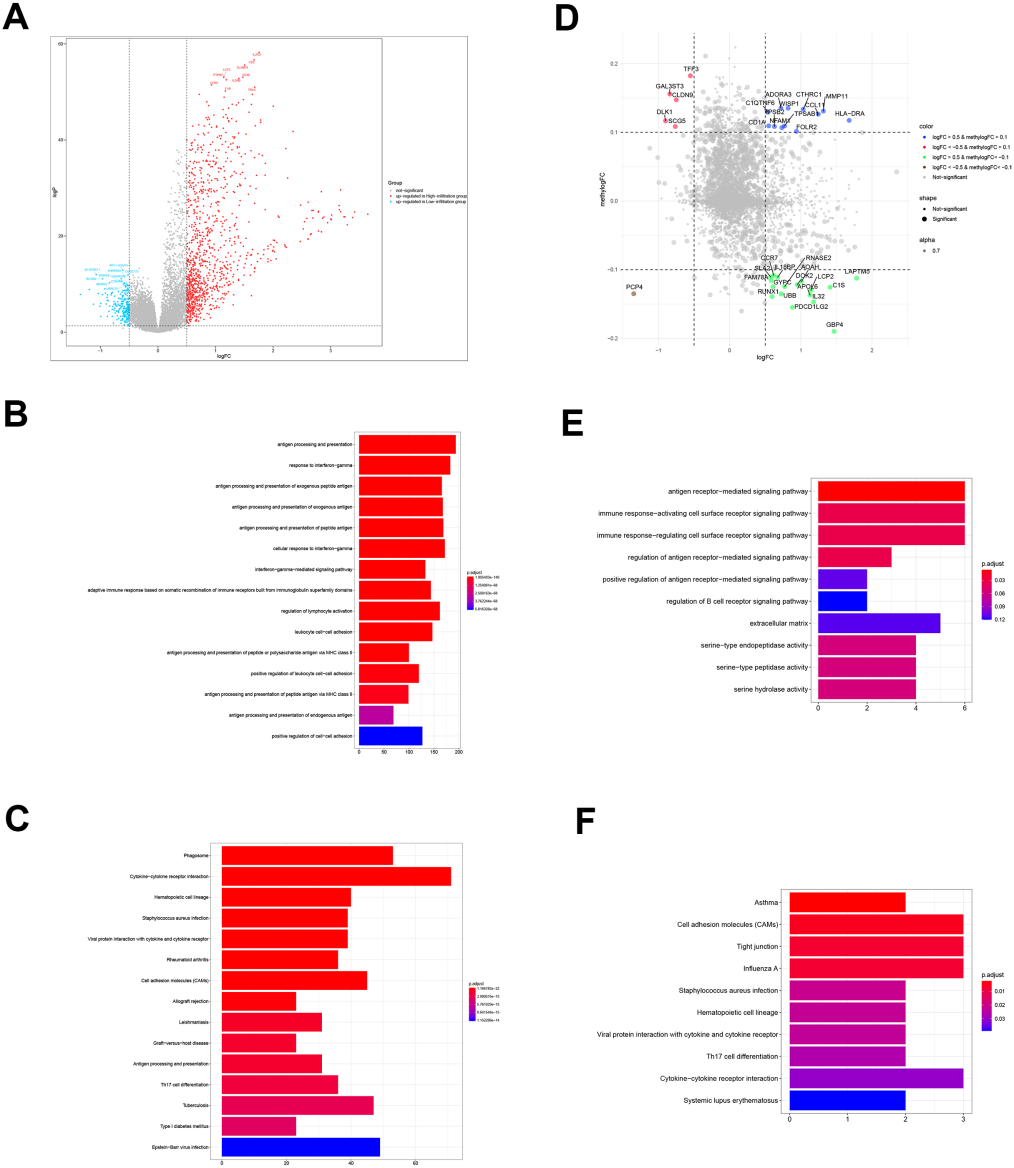
DEGs and DMGs. (A) The volcano plot shows the DEGs between TME clusters A and B. (B, C) GO and KEGG analyses of the DEGs. (D) The volcano plot shows the DMGs between TME clusters A and B. (E, F) GO and KEGG analyses of the DMGs.

To determine the biological differences between TME clusters A and B from TCGA database, we identified DEGs. Compared with TME cluster A, 494 upregulated genes and 73 downregulated genes were found in TME cluster B (Fig. 3A). The enrichment of 567 DEGs by GO and KEGG were analyzed with R cluster profiler package. DEGs from TME cluster B were observed to be enriched in immune-related pathways, mainly including the antigen presentation pathway, IFN- *γ* signaling, leukocyte activation, cytokine receptor interaction, allogeneic rejection, and Th17 cell differentiation regulation, supporting the abundant immune infiltration pattern in TME cluster B (Figs. 3B and 3C).

### Identification and functional annotation of DMGs

We performed comparative analyses to identify DMGs in ovarian cancer between TME cluster A and B using TCGA dataset. Overall, we identified 242 DMGs with 194 hypermethylated (methylogFC >0.1; adjusted *p* < 0.05) and 48 hypomethylated DMGs (methylogFC <−0.1; adjusted *p* < 0.05). We integrated the DMGs and DEGs to identify 35 differentially methylated and expressed genes (DMEGs). Furthermore, we divided the DMEGs into four different groups, namely hypo-up (*n* = 17), hyper-up (*n* = 12), hyper-down (*n* = 5), and hypo-down (*n* = 1) (Fig. 3D). We focused on the DMEGs in the hyper-down and hypo-up groups in the subsequent analyses. LAPTM5 was the most upregulated gene (logFC = 1.78, adjusted *p* < 0.001) and DLK1 was the most downregulated gene (logFC = −0.09, adjusted *p* < 0.001). Functional enrichment analyses showed that the DMGs in TME cluster B participated in important biological processes and immune related pathways, including regulation of antigen presentation, regulation of B cell receptor signaling, cytokine-cytokine receptor interaction, and Th17 cell differentiation, indicating more immune cell infiltration in TME cluster B (Figs. 3E and 3F).

### Characteristics of the immune microenvironments of the TME clusters

**Figure 4:**
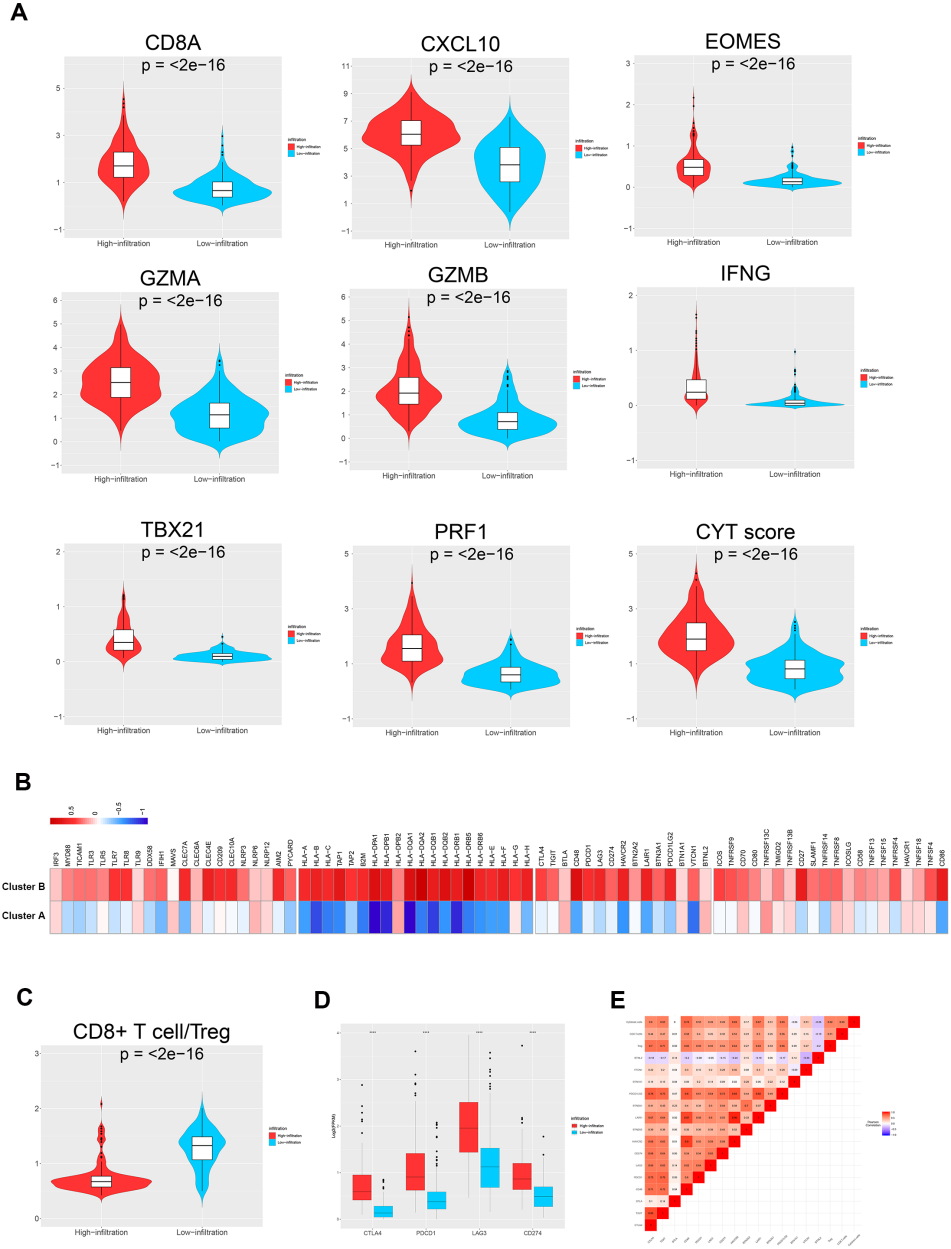
TME immune traits. (A) Violin plots showing the relative expression of the eight immune-related genes and cytolytic activity (CYT) scores. (B) Relative expression level of molecules potentially involved in initiating innate immunity (left) and those in the MHC-I/II antigen-presenting process (right). (C) Violin plots showing that the CD8+ T cells/Treg ratio was lower in TME cluster B than in TME cluster A. (D) The relative expression level of four important immune checkpoint molecules in the two TME clusters. (E) Correlation between tumor immunogenicity, immune infiltration, immune checkpoint molecules, and mutational load.

To further investigate the different genetic and molecular characteristics of TME clusters A and B, we analyzed the expression profiles of several immune-related genes and cytokines in the 343 ovarian cancer samples. First, we used a seven-gene panel derived from the Bopler trial as an alternative indicator to quantify the levels of CD8A, CXCL10, IFN- *γ*, GZMA, GZMB, EOMES, PRF1, and TBX21 (Fehrenbacher et al., 2016). These genes have previously been connected with activated T cells, immune cytolytic activity, and interferon- *γ* expression (Fehrenbacher et al., 2016; Rooney et al., 2015; Wang, Windgassen & Papoutsakis, 2008). Second, Rooney et al. (2015) defined cytolytic activity scores, namely the geometric mean of PRF1 and GZMA, to indicate the intensity of antitumor response. The parameters, including CD8A, CXCL10, EOMES, GZMA, GZMB, IFN- *γ*, TBX21, and PRF1, showed significantly higher levels in TME cluster B than TME cluster A (all *p* < 0.001), indicating that patients with higher infiltration had enhanced cytotoxic function compared with patients who showed low infiltration (Fig. 4A). The comparison of the two phenotypes (such as MyD88, TICAM1, and TLR3) with molecules potentially involved in innate immune priming showed a similar trend (except TLR9, all *p* < 0.05; Fig. 4B, left). Additionally, compared with TME cluster A, TME cluster B showed abundant expression of MHC-I/II-related antigen presenting molecules ( *p* < 0.001; Fig. 4B, right).

The TME cluster B group showed infiltration of abundant active innate and adaptive immune cells, as well as immunosuppressive cells, such as Tregs and iDCs. Therefore, we investigated the effect of immune activation and immune inhibition according to the CD8^+^ T cell/Treg ratio (Fig. 4C). This showed that TME cluster B had a significantly lower ratio than TME cluster A, which may be caused by a feedback mechanism initiated by recruiting effector T cells into the TME. In order to confirm this speculation, we investigated the expression patterns of 15 immune checkpoint molecules (Fig. 4B, left three) and 20 costimulatory molecules (Fig. 4B, right). The thermogram revealed some relatively high expressed costimulatory molecules (most *p* < 0.001, such as CTLA4, PDCD1, LAG3, and CD274, Figs. 4D and 4E) in TME cluster B group, suggesting that these patients may benefit from immune checkpoint inhibitors.

### Somatic mutations in ovarian cancer

**Figure 5:**
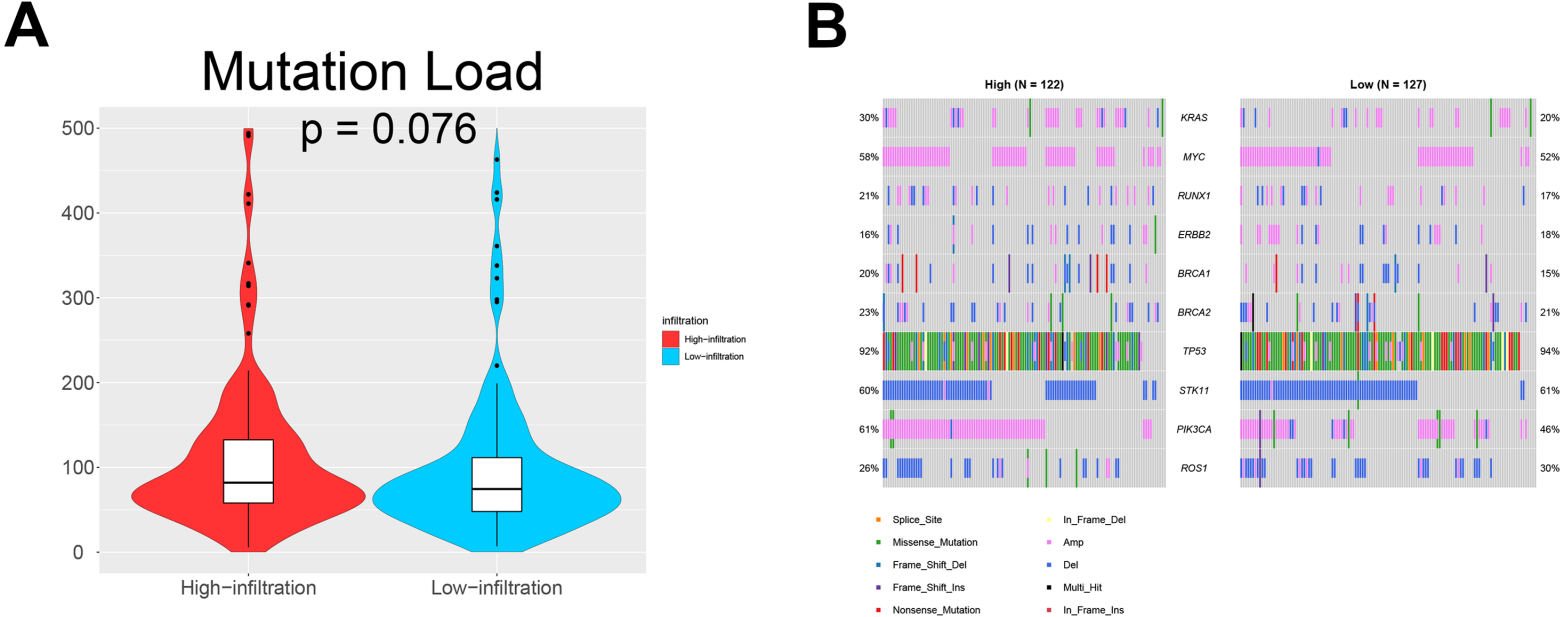
Somatic mutations in ovarian cancer. (A) Violin plots showing that the mutational load in TME cluster B was insignificantly higher compared with that in TME cluster A (*p* = 0.076). (B) Waterfall plots showing the somatic mutations in the two TME clusters.

We also investigated whether somatic mutations were associated with invasiveness independent of tumor origin. In our analyses, the mutation load in TME cluster B was insignificantly higher than that in TME cluster A (*p* = 0.076; Fig. 5A). Generally, more mutations are associated with increased heterogeneity, indicating stronger immune rejection and more prominent immune cell infiltration. We also found that patients in TME cluster B were more susceptible to PIK3CA mutation than those in TME cluster A (61% vs. 46%; *p* = 0.031; Fig. 5B). These results suggested that PIK3CA mutation may be a major driver of dysregulated expression of invasiveness-related genes in human cancer.

### Prognostic value of the TME clusters

Univariate and multivariate analyses were also performed to evaluate the relationship between several clinical factors and prognosis in the two TME clusters (Table 2). Age [hazard ratio: 0.74, 95% confidence interval (CI): 0.57–0.99; *p* = 0.046] and immune infiltration (hazard ratio: 1.33, 95%CI [1.01–1.75]; *p* = 0.041) were independent predictors of OS in ovarian cancer. Overall, TME cluster B predicted better OS than TME cluster A.

**Table 2:** Univariate and multivariate analysis of overall survival in OV patients from TCGA database.

Variable (TCGA)	Univariable		Multivariable	
	HR (95%CI)	*P*-value	HR (95%CI)	*P*-value
Age (≤65 vs. >65)	0.73 (0.56–0.98)	0.034	0.74 (0.57–0.99)	0.046
Stage				
Stage I	–	–	–	–
Stage II	0.47 (0.19–1.15)	0.097	0.13 (0.02–0.66)	0.014
Stage III	0.96 (0.69–1.34)	0.824	0.25 (0.06–1.03)	0.054
Stage VI	1.19 (0.84–1.70)	0.324	0.30 (0.07–1.27)	0.103
Infiltration (Low vs. High)	1.38 (1.05–1.81)	0.021	1.33 (1.01–1.75)	0.041

## Discussion

Recently, immunotherapy has aroused great interest following improvements in our understanding of the molecular basis of immune recognition and regulation of tumor cells. The infiltration and activation of immune cells are believed to be closely related to intrinsic tumor processes and prognosis. In ovarian cancer, it has been found that the presence of lymphocyte infiltration is associated with improved prognosis (Sato et al., 2005). In contrast, the survival rate of ovarian cancer patients who lack lymphocyte infiltration is reduced (Hwang et al., 2012). To better understand the distribution of immune cells and factors in the ovarian cancer microenvironment and find new potential targets for immunotherapy, we divided cases into a low infiltration group (TME cluster A) and an infiltration group (TME cluster B) according to the individual immune infiltration patterns. The distribution of immune cells and immune related factors were statistically analyzed. This analysis revealed that TME infiltration pattern was an independent prognostic factor for ovarian cancer patients.

DNA methylation is a major epigenetic mechanism, which plays important roles in various biological processes (Bock, 2012), including regulating gene expression (Kulis & Esteller, 2010), cell differentiation (Smith & Meissner, 2013), and inflammation (Horsburgh et al., 2015). Aberrant methylation was considered to be involved in various diseases, including ovarian cancer (Seeber & Van Diest, 2012). In this study, we identified 35 DMEGs and found that LAPTM5 was the most upregulated gene, while Dlk1 was the most downregulated gene. LAPTM5 has been reported to show a negative correlation between 5′-UTR methylation and homologous gene expression, indicating that it is at least partially regulated by DNA methylation (Cortese et al., 2008). DLK1 encodes a transmembrane protein with multiple EGF repeats that plays a role in regulating cell growth. Dlk1 overexpression participated in ovarian carcinogenesis through Notch activation and EMT induction (Huang et al., 2019), which may depend on its hypermethylated level. However, no study has focused on the relationship between Dlk1 methylation and ovarian cancer. Inhibition of RUNX1 promotes cisplatin-induced apoptosis in ovarian cancer cells (Xiao et al., 2020). MMP11 was found to be related with bowel metastases in ovarian cancer (Mariani et al., 2019). However, the methylation level of the above genes associated with their functions in ovarian cancer are still unclear.

This study had several limitations. First, we included only a small number of ovarian cancer cases. Second, we failed to use non-database cases for external verification. More clinical data are needed to verify the reliability of this classification standard. Further exploration in this area is necessary. Third, we enrolled the ovarian cancer patients with all histological subtypes because the sample size of any specific histological subtype was insufficient. In the further study, we may enroll sufficient sample size of specific histological subtypes, such as ovarian clear cell carcinoma, mucinous ovarian cancer or serous ovarian cancer, to make in-depth exploration and study.

## Conclusion

This study described the basic characteristics of the immune invasion pattern of ovarian cancer through a multi-component analysis of large-scale cohort studies, and integrated the biomarkers related to different immune phenotypes previously proposed, thus revealing the interaction between the tumor and the immune microenvironment and providing the clinical basis for providing more accurate and personalized immunotherapy strategies for ovarian cancer patients.

##  Supplemental Information

10.7717/peerj.10255/supp-1 Supplemental Information 1 Code

10.7717/peerj.10255/supp-2 Supplemental Information 2 GSE3149 series matrix

10.7717/peerj.10255/supp-3 Supplemental Information 3 TCGA-OV phenotype

10.7717/peerj.10255/supp-4 Supplemental Information 4 GSE26712 series matrix

10.7717/peerj.10255/supp-5 Supplemental Information 5 GSE63885 series matrix

## Additional Information and Declarations

### Data Availability

The following information was supplied regarding data availability: The raw measurements are available in the Supplemental Files. The raw data shows ovarian cancer patient data from TCGA (keyword: OV) and GEO (GSE3149, GSE26712, GSE63885).

## References

[ref-1] Barnholtz-Sloan JS, Schwartz AG, Qureshi F, Jacques S, Malone J, Munkarah AR (2003). Ovarian cancer: changes in patterns at diagnosis and relative survival over the last three decades. American Journal of Obstetrics and Gynecology.

[ref-2] Bi G, Chen Z, Yang X, Liang J, Hu Z, Bian Y, Sui Q, Li R, Zhan C, Fan H (2020). Identification and validation of tumor environment phenotypes in lung adenocarcinoma by integrative genome-scale analysis. Cancer Immunology and Immunotherapy.

[ref-3] Bindea G, Mlecnik B, Tosolini M, Kirilovsky A, Waldner M, Obenauf AC, Angell H, Fredriksen T, Lafontaine L, Berger A, Bruneval P, Herman Fridman W, Becker C, Pagés F, Speicher MR, Trajanoski Z, Galon J (2013). Spatiotemporal dynamics of intratumoral immune cells reveal the immune landscape in human cancer. Immunity.

[ref-4] Bock C (2012). Analysing and interpreting DNA methylation data. Nature Reviews Genetics.

[ref-5] Colaprico A, Silva TC, Olsen C, Garofano L, Cava C, Garolini D, Sabedot TS, Malta TM, Pagnotta SM, Castiglioni I, Ceccarelli M, Bontempi G, Noushmehr H (2016). TCGAbiolinks: an R/Bioconductor package for integrative analysis of TCGA data. Nucleic Acids Research.

[ref-6] Cortese R, Hartmann O, Berlin K, Eckhardt F (2008). Correlative gene expression and DNA methylation profiling in lung development nominate new biomarkers in lung cancer. International Journal of Biochemistry and Cell Biology.

[ref-7] Fehrenbacher L, Spira A, Ballinger M, Kowanetz M, Vansteenkiste J, Mazieres J, Park K, Smith D, Artal-Cortes A, Lewanski C, Braiteh F, Waterkamp D, He P, Zou W, Chen DS, Yi J, Sandler A, Rittmeyer A, POPLAR Study Group (2016). Atezolizumab versus docetaxel for patients with previously treated non-small-cell lung cancer (POPLAR): a multicentre, open-label, phase 2 randomised controlled trial. Lancet.

[ref-8] Hänzelmann S, Castelo R, Guinney J (2013). GSVA: gene set variation analysis for microarray and RNA-seq data. BMC Bioinformatics.

[ref-9] Horsburgh S, Robson-Ansley P, Adams R, Smith C (2015). Exercise and inflammation-related epigenetic modifications: focus on DNA methylation. Exercise Immunology Review.

[ref-10] Huang CC, Cheng SH, Wu CH, Li WY, Wang JS, Kung ML, Chu TH, Huang ST, Feng CT, Huang SC, Tai M-H (2019). Delta-like 1 homologue promotes tumorigenesis and epithelial-mesenchymal transition of ovarian high-grade serous carcinoma through activation of Notch signaling. Oncogene.

[ref-11] Hwang WT, Adams SF, Tahirovic E, Hagemann IS, Coukos G (2012). Prognostic significance of tumor-infiltrating T cells in ovarian cancer: a meta-analysis. Gynecologic Oncology.

[ref-12] Johnson WE, Li C, Rabinovic A (2007). Adjusting batch effects in microarray expression data using empirical Bayes methods. Biostatistics.

[ref-13] Kulis M, Esteller M (2010). DNA methylation and cancer. Advances in Genetics.

[ref-14] Lee S, Hwang KS, Lee HJ, Kim JS, Kang GH (2004). Aberrant CpG island hypermethylation of multiple genes in colorectal neoplasia. Laboratory Investigation.

[ref-15] Licchesi JD, Westra WH, Hooker CM, Herman JG (2008). Promoter hypermethylation of hallmark cancer genes in atypical adenomatous hyperplasia of the lung. Clinical Cancer Research.

[ref-16] Liu YN, Liu Y, Lee HJ, Hsu YH, Chen JH (2008). Activated androgen receptor downregulates E-cadherin gene expression and promotes tumor metastasis. Molecular and Cellular Biology.

[ref-17] Lorenzo-Sanz L, Munoz P (2019). Tumor-infiltrating immunosuppressive cells in cancer-cell plasticity, tumor progression and therapy response. Cancer Microenvironment.

[ref-18] Lowe KA, Chia VM, Taylor A, O’Malley C, Kelsh M, Mohamed M, Mowat FS, Goff B (2013). An international assessment of ovarian cancer incidence and mortality. Gynecologic Oncology.

[ref-19] Mariani A, Wang C, Oberg AL, Riska SM, Torres M, Kumka J, Multinu F, Sagar G, Roy D, Jung DB, Zhang Q, Grassi T, Visscher DW, Patel VP, Jin L, Staub JK, Cliby WA, Weroha SJ, Kalli KR, Hartmann LC, Kaufmann SH, Goode EL, Shridhar V (2019). Genes associated with bowel metastases in ovarian cancer. Gynecologic Oncology.

[ref-20] Monti S, Tamayo P, Mesirov J, Golub T (2003). Consensus clustering: a resampling-based method for class discovery and visualization of gene expression microarray data. Machine Learning.

[ref-21] Park HK, Ruterbusch JJ, Cote ML (2017). Recent trends in ovarian cancer incidence and relative survival in the united states by race/ethnicity and histologic subtypes. Cancer Epidemiology, Biomarkers & Prevention.

[ref-22] Ritchie ME, Phipson B, Wu D, Hu Y, Law CW, Shi W, Smyth GK (2015). limma powers differential expression analyses for RNA-sequencing and microarray studies. Nucleic Acids Research.

[ref-23] Rooney MS, Shukla SA, Wu CJ, Getz G, Hacohen N (2015). Molecular and genetic properties of tumors associated with local immune cytolytic activity. Cell.

[ref-24] Sato E, Olson SH, Ahn J, Bundy B, Nishikawa H, Qian F, Jungbluth AA, Frosina D, Gnjatic S, Ambrosone C, Kepner J, Odunsi T, Ritter G, Lele S, Chen Y-T, Ohtani H, Old LJ, Odunsi K (2005). Intraepithelial CD8+ tumor-infiltrating lymphocytes and a high CD8+/regulatory T cell ratio are associated with favorable prognosis in ovarian cancer. Proceedings of the National Academy of Sciences of the United States of America.

[ref-25] Seeber LM, Van Diest PJ (2012). Epigenetics in ovarian cancer. Methods in Molecular Biology.

[ref-26] Smith ZD, Meissner A (2013). DNA methylation: roles in mammalian development. Nature Reviews Genetics.

[ref-27] Tamborero D, Rubio-Perez C, Muinos F, Sabarinathan R, Piulats JM, Muntasell A, Dienstmann R, Lopez-Bigas N, Gonzalez-Perez A (2018). A pan-cancer landscape of interactions between solid tumors and infiltrating immune cell populations. Clinical Cancer Research.

[ref-28] Wang M, Windgassen D, Papoutsakis ET (2008). Comparative analysis of transcriptional profiling of CD3+, CD4+ and CD8+ T cells identifies novel immune response players in T-cell activation. BMC Genomics.

[ref-29] Xiao L, Peng Z, Zhu A, Xue R, Lu R, Mi J, Xi S, Chen W, Jiang S (2020). Inhibition of RUNX1 promotes cisplatin-induced apoptosis in ovarian cancer cells. Biochemical Pharmacology.

[ref-30] Yu G, Wang LG, Han Y, He QY (2012). clusterProfiler: an R package for comparing biological themes among gene clusters. OMICS.

